# Photodegradable Hydrogel Matrices for Spatiotemporal Control of Bacteria Transport and Delivery

**DOI:** 10.1021/acsami.5c14670

**Published:** 2025-09-02

**Authors:** Jeffrey A. Reed, Scott T. Retterer, Ryan R. Hansen

**Affiliations:** † Tim Taylor Department of Chemical Engineering, 5308 Kansas State University, Manhattan, Kansas 66506, United States; ‡ Center for Nanophase Materials Sciences, 6146 Oak Ridge National Laboratory, Oak Ridge, Tennessee 37830, United States

**Keywords:** hydrogels, biotherapeutics, living materials, photodegradation, chemotaxis, bacteria, poly(ethylene glycol)

## Abstract

Stimuli-responsive hydrogels that provide controlled degradation can be used as bacteria delivery systems for advanced therapeutic applications. Here, we report the first use of photodegradable hydrogels as materials that can direct bacterial movement, tune mean bacteria speed, and control bacteria delivery through spatiotemporal control of degradation. Hydrogels were formed using base-catalyzed Michael addition reactions between photodegradable poly­(ethylene glycol) (PEG) *o*-nitrobenzyl diacrylate macromers and PEG tetra-thiol cross-linkers within microfluidic channels. Nutrient gradients were generated across the channel, and micron-scale regions of the hydrogel were partially degraded by exposure to controlled doses (2.1–168 mJ/mm^2^) of patterned 365 nm light. Hydrogel degradation was then characterized *in situ* using fluorescence visualization of fluorescein-labeled hydrogels. Following characterization, *Bacillus subtilis* expressing green fluorescent protein was introduced into the device, and its movement up the nutrient gradient was monitored using time-lapse fluorescence microscopy to enable a systematic study of bacteria chemotaxis through the hydrogels at varied levels of degradation. *B. subtilis* showed minimal adhesion to partially degraded PEG hydrogels, and bacteria mean speed and mean directional change were tunable according to the level of hydrogel photodegradation, with a 2.6-fold difference in mean cell speed measured across the partially degraded hydrogel regions. Finally, the ability to alter bacteria speed and directionality through tunable degradation and without significant adhesion was used to achieve controlled release profiles of bacteria to delivery sites. These findings advance the use of PEG-based hydrogel materials as delivery vehicles for bacterial therapeutic applications and other living material applications that require controlled bacteria transport.

## Graphical abstract

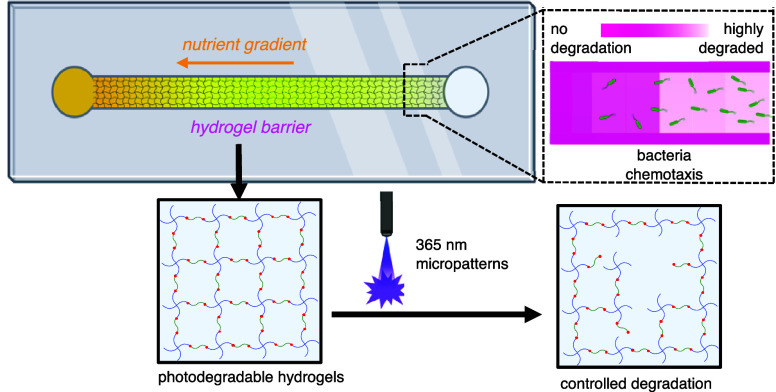

## 1 Introduction

Bacteria can be used to treat human diseases, including cancer, gastrointestinal diseases, and metabolic disorders. For example, anticancer bacteria can destroy malignant tissues through exotoxin secretion, nutrient consumption, or immune cell activation, and can be engineered to carry therapeutic payloads and release them within the tumor environment.^1,2^ Further, bacteria can be engineered to sense and respond to biochemical cues from diseased cells, enabling chemotaxis to the disease site.^3^ However, a critical limitation is achieving targeted delivery of viable bacteria cells in a controlled manner to ensure efficacy while avoiding an immune response or pathogenic side effects.^4^ Hydrogel encapsulation for delivery is a promising solution, as these materials can provide a protective environment for bacteria and have the potential for controlled and localized release. Bacteria-encapsulated hydrogels have been demonstrated to improve efficacy and safety, particularly for probiotic treatments of gastrointestinal disorders.^5,6^ While the majority of hydrogels used to encapsulate bacteria consist of naturally occurring polysaccharide-based materials (alginate, chitosan, starch, etc.), encapsulation in synthetic hydrogels offers significant advantages. This includes design for response to environmental stimuli such as temperature, pH, and ionic strength for triggered and controlled release of cargo into a targeted environment.^7^ While synthetic, stimuli-responsive hydrogels have found extensive use in biomolecular drug delivery,^8,9^ they are currently underutilized in bacteria therapeutic applications.

Poly­(ethylene glycol) (PEG)-based hydrogels hold unique advantages for bacteria encapsulation and delivery, as PEG is chemically and biologically inert and nonadhesive to bacteria,^10,11^ covalently cross-linked to provide stability, and can be chemically modified to generate hydrogels that change physicochemical properties in response to a variety of stimuli. Further, PEG is one of the most versatile and adaptable biomaterials, with commercially available macromers available across a wide range of molecular weights for precise control of hydrogel pore sizes and mechanical properties. PEG is also available with a variety of end-group chemistries for selection of the polymerization chemistry used for encapsulation. Chain-growth polymerizations are commonly used for PEG hydrogel generation, but have drawbacks when used for cell encapsulation that include the generation of free radicals and hydrogel network heterogeneities.^12,13^ Step-growth polymerizations that use click chemistry avoid many of these limitations, as they can provide near-complete conversion of cross-linker molecules, do not require external initiators in some cases, do not generate reaction byproducts or free radicals,^14^ and form hydrogels with less network heterogeneity at all length scales for higher control of mass transport.^15–19^ Given these advantages, we have recently used thiol-Michael addition reactions to encapsulate bacteria in PEG, demonstrating that these chemistries offer high cell viability and provide a stable matrix for 3D bacteria culture and control of mass transfer, making them useful as protective coatings against environmental toxins.^20,21^

Light holds unique advantages as a stimulus for bacteria-hydrogel systems, as it allows for spatiotemporal manipulation of bacteria cells. For example, Matsumoto et al. designed photoresponsive glycolipids that undergo reversible gel–sol/sol–gel transitions with light exposure to spatially localize bacteria.^22^ In recent years, we have added the photocleavable moiety *o*-nitrobenzyl (*o*-NB) to PEG hydrogels to yield photodegradable hydrogels that allow for spatial degradation with patterned light for selection and isolation of bacteria from heterogeneous cell mixtures,^23–25^ a key capability in the development of microwell arrays that screen and select bacteria with unique function from diverse microbial communities.^26–28^ While 365 nm light was used here for degradation, this can be transitioned to near-infrared light with addition of up-conversion nanoparticles to these hydrogels to improve cytocompatibility and enable *in vivo* delivery applications.^29^

Motivated by the need to develop materials that can control the release profile of bacteria for therapeutic efficacy,^4^ this study provides a systematic investigation of bacteria transport through PEG-based hydrogel materials at different stages of degradation in effort to control bacteria movement and release. Photodegradable thiol–acrylate polymerized PEG hydrogels are deposited within a microfluidic device and subjected to controlled levels of degradation using varied light doses. A nutrient gradient is then generated across the hydrogels to promote bacterial chemotaxis through the hydrogel. The uniform network structure of these hydrogels,^16^ combined with spatiotemporal control of degradation and microfluidic control of the chemical environment, enables a highly systematic experimental approach. *Bacillus subtilis* was chosen as the focal species due to its unique potential in bacterial therapeutic applications.^30^ This includes its designation as a generally recognized as safe (GRAS) bacterium, its ability to sporulate for long-term stability, its ease of genetic modification for drug delivery, and its natural production of bioactive secondary metabolites such as surfactin, with known antimicrobial and anticancer properties.^31^ The results reveal that tunable degradation of PEG-based hydrogels can be used to control the speed, directionality, and release profile of bacteria, findings directly applicable to therapeutic bacteria delivery. Beyond biotherapeutics, this study broadly informs other engineered living material applications that rely on controlled microbial transport in hydrogels,^32^ as well as antifouling approaches that employ PEG-based hydrogels with the prerequisite of inhibiting bacterial infiltration,^33,34^ and bioinoculant applications,^35^ where hydrogel-microfluidic systems can serve as well-controlled porous-media analogs to understand microbial transport in soils.^36–38^

## 2 Results and Discussion

### 2.1 Concept and Device Characterization

The strategy used to probe bacteria taxis through the partially degraded PEG hydrogels involved the deposition and patterned degradation of the hydrogel within a microfluidic channel (Figure 1). Microfluidic devices were designed to contain a single channel (width = 250 μm, height = 8.3 μm, length = 3000 μm) between a nutrient-rich media source and a nutrient-deficient sink well. The hydrogel was deposited by mixing the PEG tetrathiol (*M*_w_ 5000 Da) and PEG-*o*-nitrobenzyl diacrylate (PEG-*o*-NB-DA, *M*_w_ 3400 Da) macromers together in basic buffer (pH 8.0), then quickly loading this solution within the microchannel for formation of photodegradable PEG hydrogels (herein referred to as *pd*-PEG hydrogels), driven by thiol–acrylate addition cross-linking reactions. This precursor solution was developed and characterized in prior studies.^24,39^ It generates an average mesh size of 10 nm when fully swollen and has a gelation time of ∼25 min, providing adequate time to load the liquid precursor solution within the microchannel. After hydrogel formation, addition of nutrient-rich media into the “source” well and a nutrient-deficient solution into the “sink” well on either side of the channel generated a chemical driving force for passive nutrient diffusion, forming a nutrient gradient across the hydrogel. Subsequent exposure with user-defined, micron-scale light patterns enabled spatially controlled degradation of the hydrogel within the desired region of interest (ROI) at the end of the channel. Inoculation of a dilute suspension of *B. subtilis*-GFP into the nutrient-deficient sink positions the cells in an environment that promotes chemotaxis across the hydrogel barrier.

**Figure 1:**
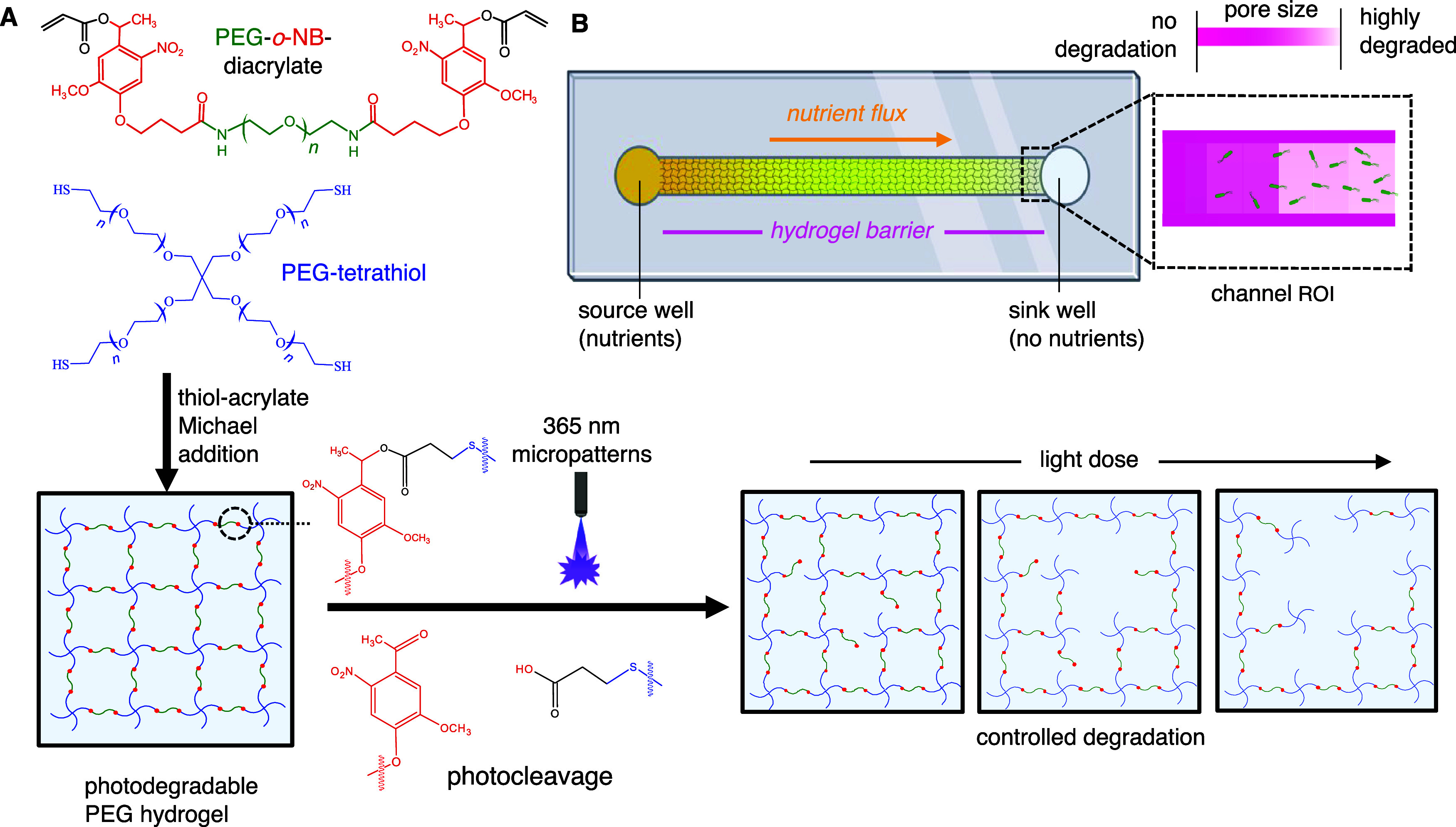
(A) Formation of *pd*-PEG hydrogel using a base-catalyzed Michael-addition reaction between PEG-*o*-NB-diacrylate and PEG-tetrathiol and photocleaving mechanism using micropatterned 365 nm light. (B) Microfluidic device overview, including chemical gradient generation across a hydrogel barrier and the channel region of interest, where all photodegradation and chemotaxis experiments were performed. Panel (B) was completed in BioRender software.

To visualize chemical gradient formation within the microchannel, an Alexa Fluor dye was added to the source well to mimic nutrient diffusion across the hydrogel. 20× fluorescent image montages of the channel from 1 to 48 h show the evolution of the chemical gradient (Figure 2A,B). It is important to note that camera settings were chosen to quantitatively track chemical gradient formation within the ROI at the end of the channel; thus, the fluorescent signal from the left side of the channel in Figure 2A becomes saturated at later time points when fluorophore concentrations become high. The transient chemical gradient enabled quantification of the effective diffusion coefficient (*D*_eff_) through the hydrogel using the early time approximation to Fick’s Second Law (Figure 2C). This was calculated to be *D*_eff_ = 2.7 × 10^–7^ ± 1.5 × 10^–7^ cm^2^/s, which is a 88% decrease relative to Alexa Fluor 594 diffusivity in water (2.3 × 10^–6^ cm^2^/s),^40^ indicating hindered diffusion throughout the hydrogel. The decrease is comparable to drops in diffusivities measured for other small molecules in similar step-growth PEG hydrogels of comparable mesh sizes (ex. 87% reduction of acetate diffusivity).^20^ This indicates that convective flows are minimal or absent across the channel, as convective mass transport in the positive *x*-direction would lead to an artificially high diffusion coefficient, while convective transport in the negative *x*-direction would inhibit chemical gradient formation. After 24 h, a linear concentration gradient was noted in the second half of the channel and remained stable through 48 h (Figure 2B), indicating the device reached a pseudosteady state during this period. To extend these findings to the diffusion of actual nutrients present in tryptic soy broth (TSB) media (glucose, tryptone) for chemotaxis experiments, a mass transfer model that assumes a similar 88% reduction in glucose and tryptone diffusivities was developed (described in Supporting Information, Section S1.0). The model predicts steady-state nutrient concentration profiles after 24 h (Figures S1–S3). For this reason, devices were incubated for 24 h prior to introduction of cells to enable formation of a steady nutrient gradient. Subsequent chemotaxis experiments occurred during the 24–48 h period in the channel ROI (*x*/*L* = 0.8–1.0, Figure 2A,B).

**Figure 2:**
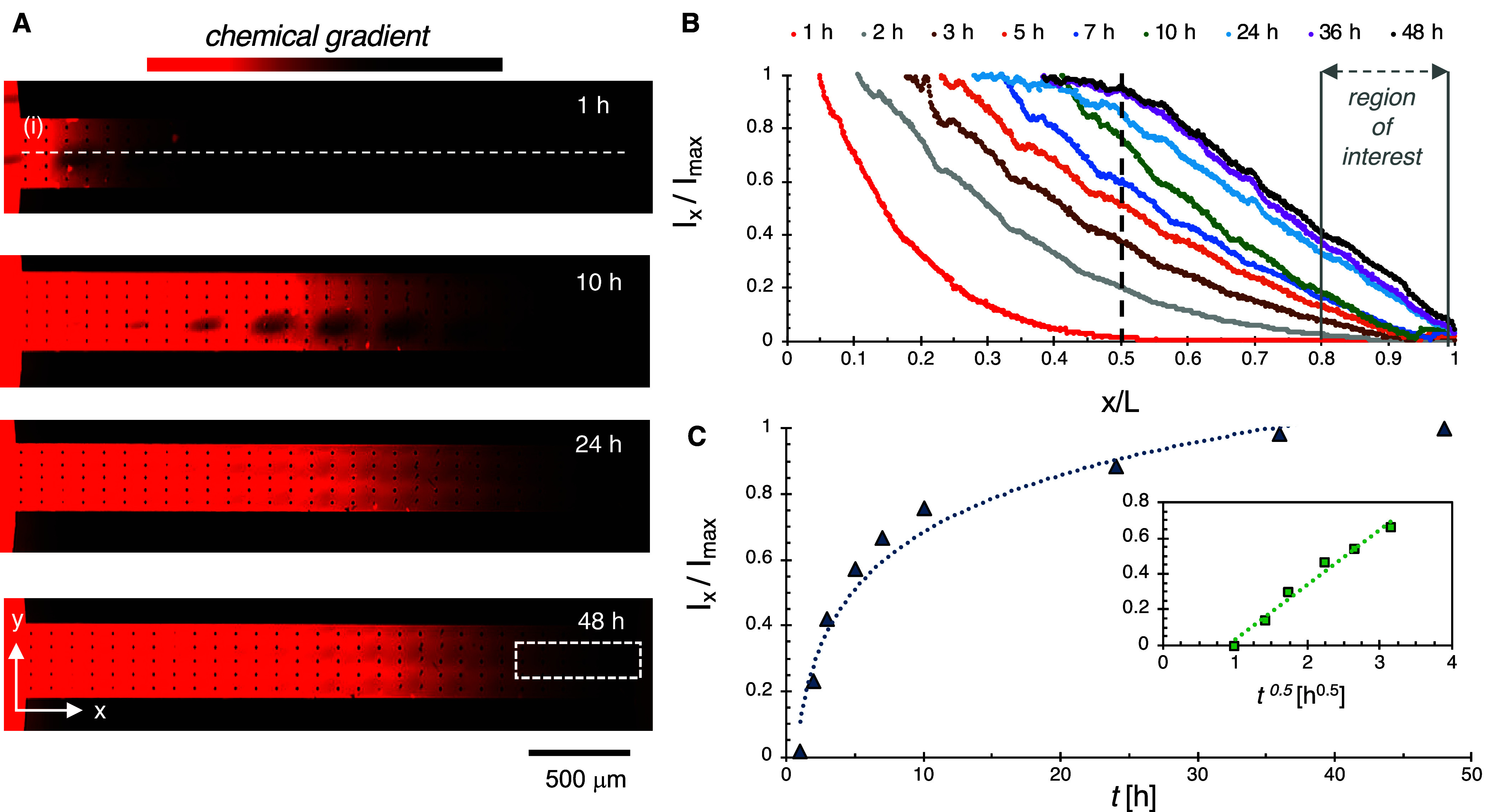
(A) 20× Image montages of the microfluidic channel at 1, 10, 24, and 48 h after hydrogel formation and loading of Alexa Fluor 594 into source well. Note: Dark marks observed on 1 and 10 h are imaging artifacts. The white rectangle (48 h) corresponds to the region of interest used in all experiments. (B) Fluorescent intensity profiles of the microfluidic channel at 1, 2, 3, 5, 7, 10, 24, 36, and 48 h. Intensities and positions are normalized with respect to maximum intensity (*I*_max_) and channel length (*L* = 3000 μm), respectively and only quantitative signal data is displayed. (C) Transient fluorescence intensities taken at *x*/*L* = 0.5 (corresponding to dashed vertical black line in (B)) and (inset) early time approximation (*I*_*x*_/*I*_max_ < 0.6) used to determine *D*_eff_ for Alexa Fluor 594 through the hydrogel.

Next, to confirm the attraction of *B. subtilis*-GFP cells to the nutrient source, devices that contained *pd*-PEG hydrogels were fabricated, and a small square (140 μm × 140 μm) between the ROI and sink well was formed by exposing the hydrogel to a patterned square exposure with a 168 mJ/mm^2^ dose, which was previously characterized to cause reverse gelation in the *pd*-PEG hydrogels.^24^ This provided an imaging region in the ROI for free swimming bacteria against a fully intact hydrogel barrier. A sharp hydrogel–liquid interface corresponding to the patterned region was observed in brightfield images after degradation (Figure 3A). Next, a nutrient solution (TSB) was added into the source well instead of Alexa 594. As a negative control, 1× PBS was loaded into the source well of a separate device. After 24 h, *B. subtilis*-GFP cells were introduced to the sink well and the ROI was monitored. Within 1 h, cells were found congregating at the hydrogel–liquid interface when the nutrient gradient was present (Figure 3B,C). When the nutrient gradient was absent (Figure 3D,E), cells did not appear at the hydrogel–liquid interface and instead remained dispersed throughout the sink well. Comparing the number of cells present in the 140 μm × 140 μm imaging region with and without a nutrient gradient (Figure 3F) confirmed that cellular attraction within the ROI was due to nutrient diffusion across the hydrogel, and not due to passive attachment and aggregation of *B. subtilis* cells at the hydrogel interface. Critically, cell movement was inhibited at the sharp interface between fully degraded and fully intact hydrogel (Figure 3B and Supp. Mov. 1), indicating that bacteria were unable to penetrate the fully intact hydrogel despite the nutrient gradient. These results validated the device design, as both patterned hydrogel photodegradation within the device and a positive responsive from *B. subtilis* to the nutrient gradient generated across the hydrogel were achieved.

**Figure 3:**
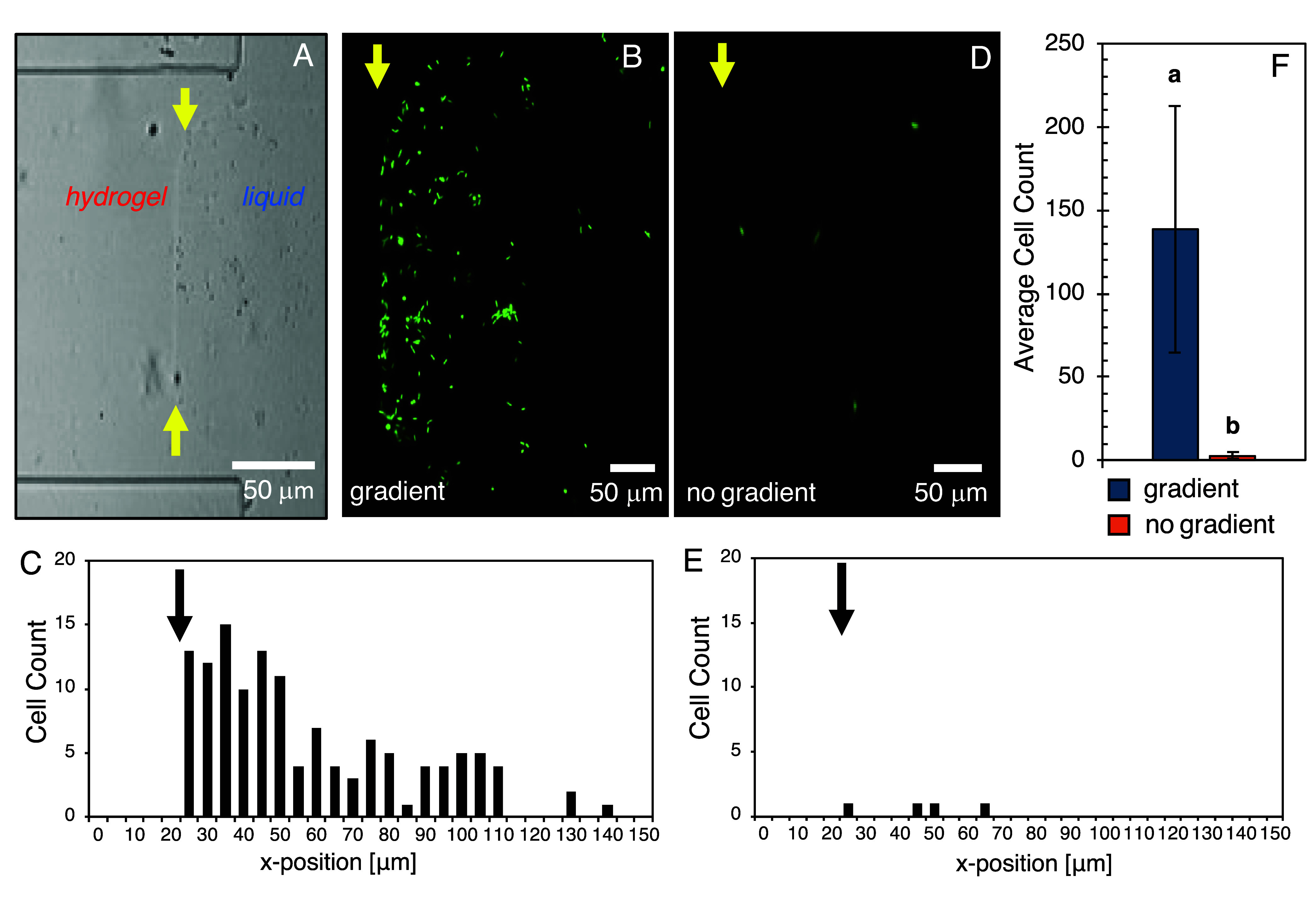
(A) Brightfield image of the ROI in the microfluidic device after bacteria loading, showing degraded/undegraded hydrogel. (B, C) Cell count and position of bacteria cells in the ROI region with a nutrient gradient, and (D, E) no nutrient gradient. (F) Average cell count of bacteria in the degraded hydrogel region with and without a chemical gradient (*N* = 3). Letters indicate statstical significance with *F*-value 10.0 and *p*-value <0.05.

### 2.2 Controlled Hydrogel Photodegradation within the Channel

As the cross-linking density of *pd*-PEG hydrogels can be systematically decreased by tuning the dose of 365 nm irradiation, the next step was to use micropatterned light to partially degrade specific areas of the hydrogel within the channel ROI while avoiding reverse gelation. The channel ROI was first exposed to a square UV pattern to reverse gelation (*t*_exp_ = 40 s, dose = 168 mJ/mm^2^), forming a region for free-swimming bacteria within the channel next to the sink well. Subsequent sections of the hydrogel were then patterned with 110 μm × 101 μm rectangles with decreasing exposure times of 10, 3, 1, 0.5 s at constant light intensity, corresponding to light doses of 42, 12.6, 4.2, and 2.1 mJ/mm^2^, forming a degradation ladder (Figures 4A and S4A,B). The ladder pattern was designed to provide a comparative analysis of cell movement immediately outside the hydrogel (fully degraded region) with cell movement at specified levels of degradation. As preliminary experiments had shown that *B. subtilis* could not move through hydrogels after a 2.1 mJ/mm^2^ dose, this was chosen as the lowest dose. Importantly, undegraded regions of hydrogel were left between the exposed region and the vertical microfluidic channel wall to enable hydrogel swelling in the *y*-direction.

**Figure 4:**
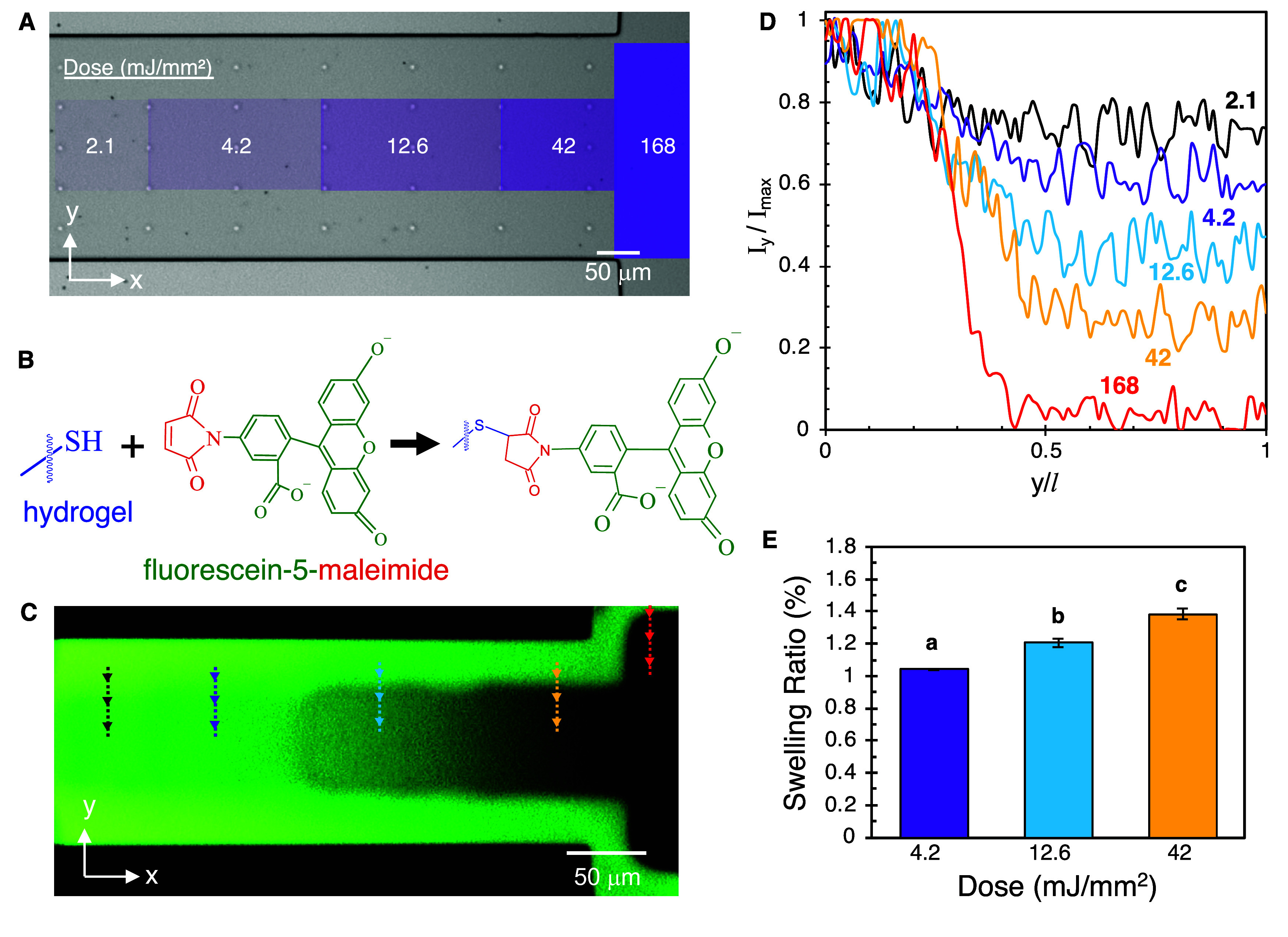
(A) Light exposure map for forming degradation ladder. (B) Chemical coupling of fluorescein-5-maleimide to pendant thiols on intact hydrogel. (C) Image of fluorescein stained hydrogel after exposure according to map in (A). (D) Fluorescent intensity profiles through degraded regions of hydrogel at each specified dose (mJ/mm^2^). Data correspond to the vertical line scans of length *l* shown in C. Arrows in line scans denote the direction and start from undegraded hydrogel (y/*l* = 0) and move into a degraded hydrogel region. *I*_max_ was taken at the beginning of the line scan (y/*l* = 0). (E) Swelling ratio, taken as the ratio of measured width after swelling to the width of the light exposure pattern (101 μm) for each exposure region (*n* = 3), Letters indicate statistical significance with *p*-value <0.01.

It was necessary to characterize the regions of partially degraded hydrogels prior to chemotaxis studies. Because the hydrogel is spatially confined between the glass and the PDMS microfluidic channel, hydrogel swelling for pore size expansion after degradation is restricted. Therefore, *in situ* characterization of each partially degraded hydrogel region within the device was necessary. With a limited number of experimental methods available for this characterization, a fluorescent staining method utilizing fluorecein-5-maleimide that we previously developed for visualizing PEGDA–PEGTT hydrogels was adapted.^23^ Covalent coupling of fluorecein-5-maleimide occurs through rapid, specific Michael addition between maleimide groups and pendant thiol groups within the hydrogel in a manner proportional to PEG chain density, enabling a semiquantitative analysis of degradation levels within the hydrogel ROI (Figure 4B). After fluorecein-5-maleimide perfusion through the partially degraded hydrogels, fluorescence imaging revealed the degradation pattern (Figure 4C). Some spatial variation in fluorescence intensity appeared within nondegraded portions of the hydrogel in the *x*-direction, indicating that there was some nonuniformity in the stain, however fluorescence intensity traces in the *y*-direction were uniform (Figure S5). Thus, normalized intensity line plots in the *y*-direction were plotted and compared across each exposure region (Figure 4C,D) and indicate that proportional decreases in PEG chain densities were found in partially degraded hydrogels with increasing UV exposure times, demonstrating patterned and controlled degradation within the channel ROI.

Imaging also revealed structural changes of the hydrogel within the channel. Degradation and lowered cross-linking densities cause an influx of water into the partially degraded hydrogel regions, resulting in swelling that is inversely proportional to cross-linking density.^41^ Within the microfluidic channel, the hydrogel is confined between the glass surface at the device floor and the relatively rigid poly­(dimethylsiloxane) (PDMS) elastomer (*E*_PDMS_ = 1.9 × 10^6^ Pa),^42^ constraining swelling in the *z*-direction. However, because a region of undegraded hydrogel was intentionally left between the pattered region and the channel wall (Figure 4A), hydrogel swelling occurs in the *y*-direction to compress the unexposed regions of fully intact hydrogel, which is softer than the PDMS walls (*E*_hydrogel_ ∼ 1.0 × 10^4^ Pa).^16^ This enabled quantification of a hydrogel swelling ratio, defined here as the width of the swollen hydrogel divided by the width of the exposed region (101 μm). Consistent with fluorescent intensity plots, increased swelling ratios with higher dose were noted after exposure and equilibrium swelling for 4.2, 12.6, and 42 mJ/mm^2^ (Figure 4E), no differences were found at 2.1 mJ/mm^2^. Taken together, these results indicate that doses between 4.2 and 42 mJ/mm^2^ reduced the cross-linking density without causing reverse gelation, causing hydrogel swelling and increased pore sizes within the channel in a manner proportional to exposure time.

### 2.3 Chemotaxis of *B. subtilis* through Partially Degraded Hydrogels

To observe bacteria chemotaxis throughout the partially degraded hydrogel regions, *B. subtilis*-GFP cells were introduced into the sink well after nutrient gradient formation and photodegradation. Bacteria cells moved throughout each rectangular region and up the nutrient gradient, with most cells accumulating at the cell impermeable interface located between the 2.1–4.2 mJ/mm^2^ doses, as cells were unable to penetrate further into the 2.1 mJ/mm^2^ exposed region (Figure 5A and Supp. Mov. 2). Some cells also congregated at the interface of partially degraded and fully intact, impermeable hydrogel, suggesting that the partial degradation pattern also generated a nutrient gradient in the *y*-direction. Importantly, very few cells remained attached within the central regions of the partially degraded hydrogels, and there was no trend in cell immobilization with PEG density or degradation level across these central regions (Figure 5B). While the few immobilized cells in the central regions may also be in contact with the microfluidic device floor (glass) or ceiling (PDMS), this observation indicates that PEG chains do not cause significant bacterial adhesion, as expected. This demonstrates PEG as an ideal hydrogel material for facilitating bacteria transport while minimizing bacteria adhesion.

**Figure 5:**
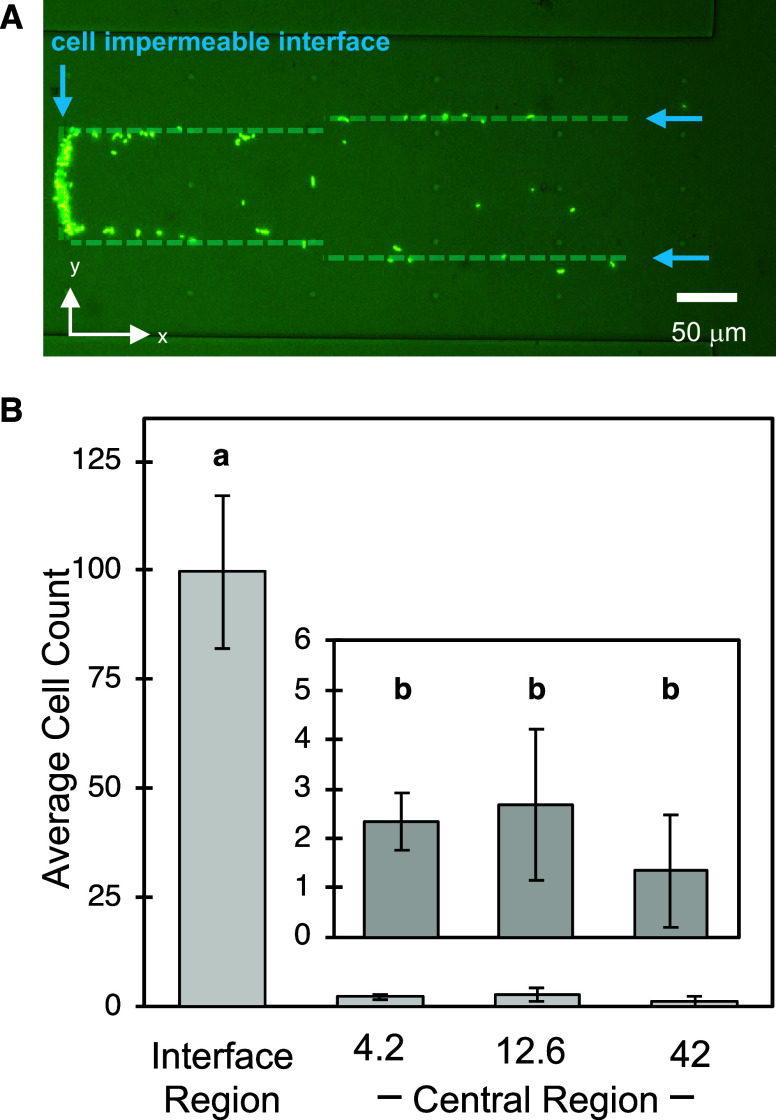
(A) Image of *B. subtilis*-GFP cells during chemotaxis through the hydrogel ladder. Blue arrows and dashed lines indicate the interface between cell-permeable and cell-impermeable hydrogels, where cell accumulation was observed. (B) Comparison of the number of cells immobilized at the cell impermeable interface regions with the number of cells immobilized within the central region of the 4.2, 12.6, and 42 mJ/mm^2^ dose zones (*n* = 3). Letters indicate statistical significance with *p*-value <0.05.

Cell movement in each region of degradation was analyzed using TrackMate software to generate individual two-dimensional cell trajectories and corresponding cell migration maps that show cell densities after 24 h (Figure 6A–D). The trajectories allowed for the mean speed and mean directional change of bacteria populations moving through each region to be quantified in each region of degradation (Figure 7). Without hydrogel confinement, free-swimming cells were observed within the 168 mJ/mm^2^ light dose region, where hydrogel reverse gelation occurred to generate a liquid phase. Here, *B. subtilis* cells display characteristic run-and-tumble motility patterns and have the highest mean speed and the highest directional change (Figures 6A and 7). The cell migration map indicates net migration toward the nutrient gradient, with significant movement in the *y*-direction. In partially degraded hydrogels exposed to higher doses (42 and 12.6 mJ/mm^2^ dose), cells experienced proportional decreases in mean speed, and also a significant drop in mean directional change as cells lost their run-and-tumble motility due to confinement within the hydrogel. Trajectories became smoother and more directed toward the nutrient gradient, causing cells to migrate further toward the nutrient source (Figure 6B,C). Mean cell speed was again reduced proportionally when moving through the region of lowest degradation (4.2 mJ/mm^2^ dose, Figure 7A). Here, cell mean directional change increased significantly compared to the two higher doses that also generated partially degraded hydrogels (Figure 7B), which indicates that cells had to find and follow more tortuous paths to move throughout this region. This caused less net migration of cell populations toward the nutrient gradient (Figure 6D).

**Figure 6:**
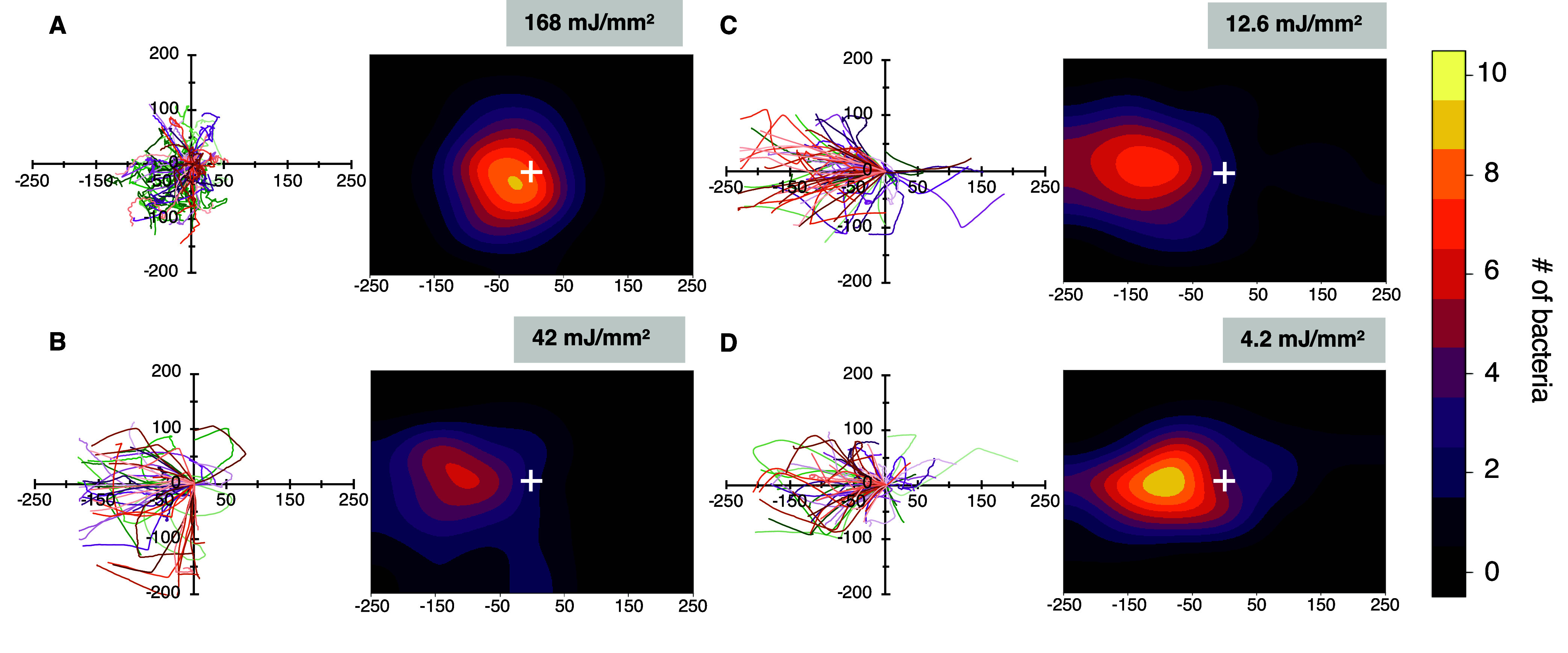
Individual two-dimensional cell trajectories tracks (left) and corresponding two-dimensional cell migration maps (right) for (A) 168 mJ/mm^2^, (B) 42 mJ/mm^2^, (C) 12.6 mJ/mm^2^, and (D) 4.2 mJ/mm^2^ exposure regions within the hydrogel. The white cross at the center of each map indicate the initial center of mass for each cell population.

**Figure 7:**
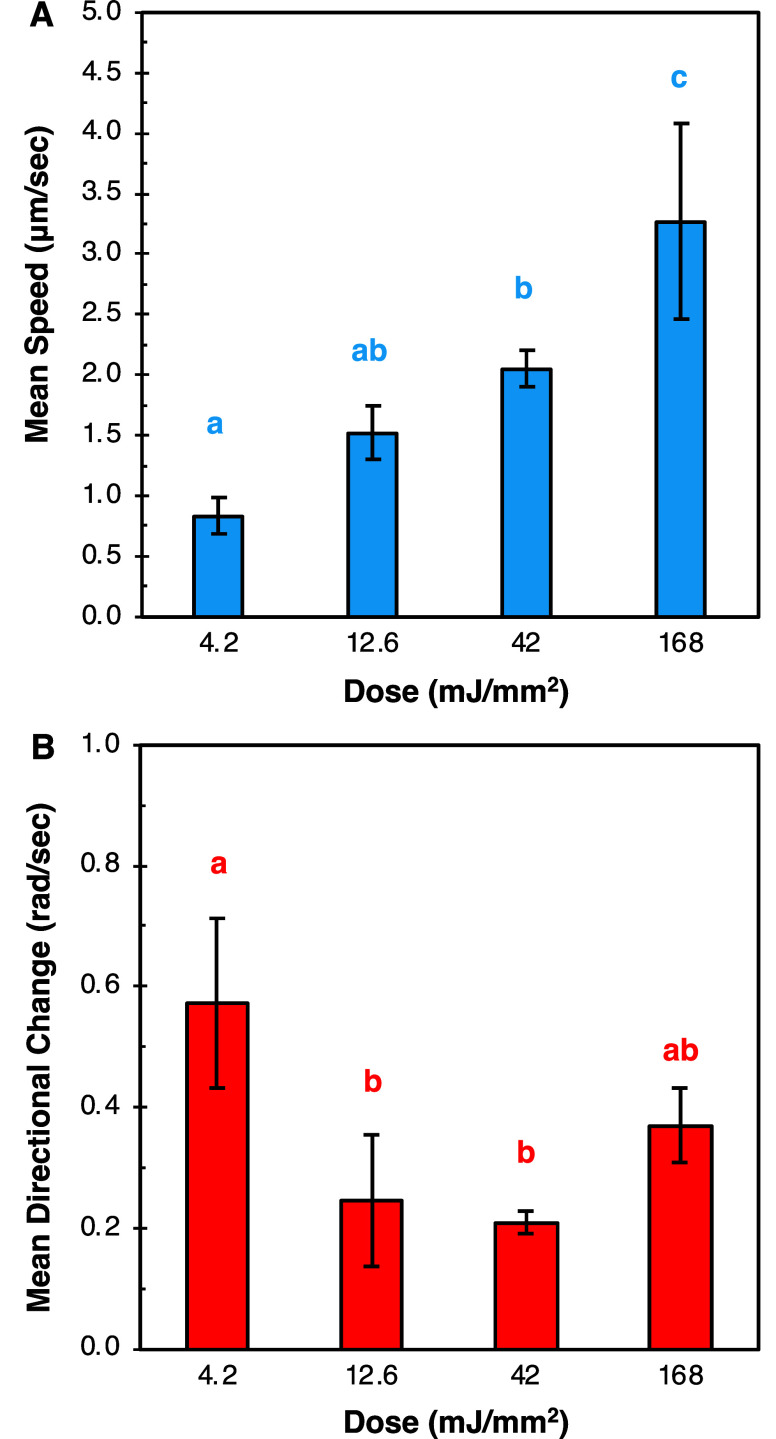
(A) Mean speed and (B) mean directional change calculated using TrackMate. An average of 73 ± 4 cells were measured for speed and mean directional change calculations for 4.2, 12.6, and 42 mJ/mm^2^ doses and 261 cells were measured for 168 mJ/mm^2^ dose. Doses of 4.2 to 42 mJ/mm^2^ correspond to partially degraded gels, 168 mJ/mm^2^ correspond to free swimming cells in a fully liquid region. Cells were evaluated over *n* = 3 independent experiments. Letters indicate statistical significance with *p* <0.05.

Interestingly, during observation of cell movement in real-time, cells in regions of lower degradation could occasionally be found traveling in identical paths toward the chemical gradient at higher speeds (noted example on dashed white line in Supp. Mov. 2). While a qualitative observation only, this led to the hypothesis that cells could find and exploit “paths of least resistance” within the partially degraded hydrogel to quickly advance toward the nutrient gradient. To further explore this hypothesis, an interface between a fully intact hydrogel and a fully degraded liquid region was again developed. Pathways of compromised hydrogel were then introduced at the liquid-hydrogel interface using three narrow line patterns of 9 μm width, 208 μm length at different light doses (21, 42, 63 mJ/mm^2^), offering pathways of compromised hydrogels at varied levels of degradation. After introduction into the device, *B. subtilis* cells were able to find and penetrate through these compromised pathways (Figure S6 and Supp. Mov. 3) to progress up the nutrient concentration gradient. Here, cell trajectories became increasingly linear with lower dose (Figure S6C–E), as cell motion was directed toward two-dimensional paths due to less degradation and swelling. These trends indicate that bacteria find and exploit “paths of least resistance” when penetrating through partially degraded hydrogels.

### 2.4 Selective and Controlled Delivery of Live Bacteria with Partially Degraded Hydrogels

A comparison of the averaged cell speeds at different light doses (Figure 7) indicates that controlled degradation can be used to tune *B. subtilis* speed down to nearly 25% of its free-swimming speed. This suggests that this material could be used to control the release and accumulation of bacteria to a targeted location according to its degradation level. To demonstrate this, the degradation pattern within the microfluidic channel was modified to include a fully degraded, liquid region next to the sink well (168 mJ/mm^2^ exposure), followed by a partially degraded region (220 μm length × 101 μm width) and finally, a fully degraded “delivery” region (110 μm length by 101 μm width) to collect the bacteria released from the partially degraded hydrogels (Figures 8A and S4C). To verify that active, motile bacteria were required to reach the delivery region, nonactive red fluorescent polystyrene beads of similar size (1 μm diameter) were mixed with *B. subtilis* at a 10:1 bead/cell ratio (Figure S7) in the sink well. Degradation levels within the partially degraded region were tuned using the three different doses (42, 12.6, or 4.2 mJ/mm^2^), providing increasing resistance to molecular and cellular transport. After loading the bacteria-bead mixture into the well, the hydrogel was monitored (Supp. Mov. 4, 5, and 6), and the cell and bead counts accumulating in the delivery region were quantified. Over 10 h, cells accumulated in the delivery region at higher numbers when more degradation was provided to the partially degraded region (Figure 8B,C). Statistically significant differences in cell counts were measured between 4.2 and 42 mJ/mm^2^ degradation at later time points (*p* ≤ 0.05 at 10 h). The differences in the number of cells released into the delivery region reflect the differences in mean swimming speeds observed at the different exposure doses (Figure 7A). Additionally, despite the 10-fold excess of beads in the sink well, no significant accumulation of beads were measured in the delivery region during the 10 h period (Figure 8C, inset). This indicates that cellular motility, as opposed to passive diffusion or convective transport, was required for cells to reach the delivery region. Overall, the results demonstrate that tuning the hydrogel cross-linking density through controlled degradation can be exploited to control the release profile of motile bacteria, an important need for therapeutic bacteria delivery.^4^

**Figure 8:**
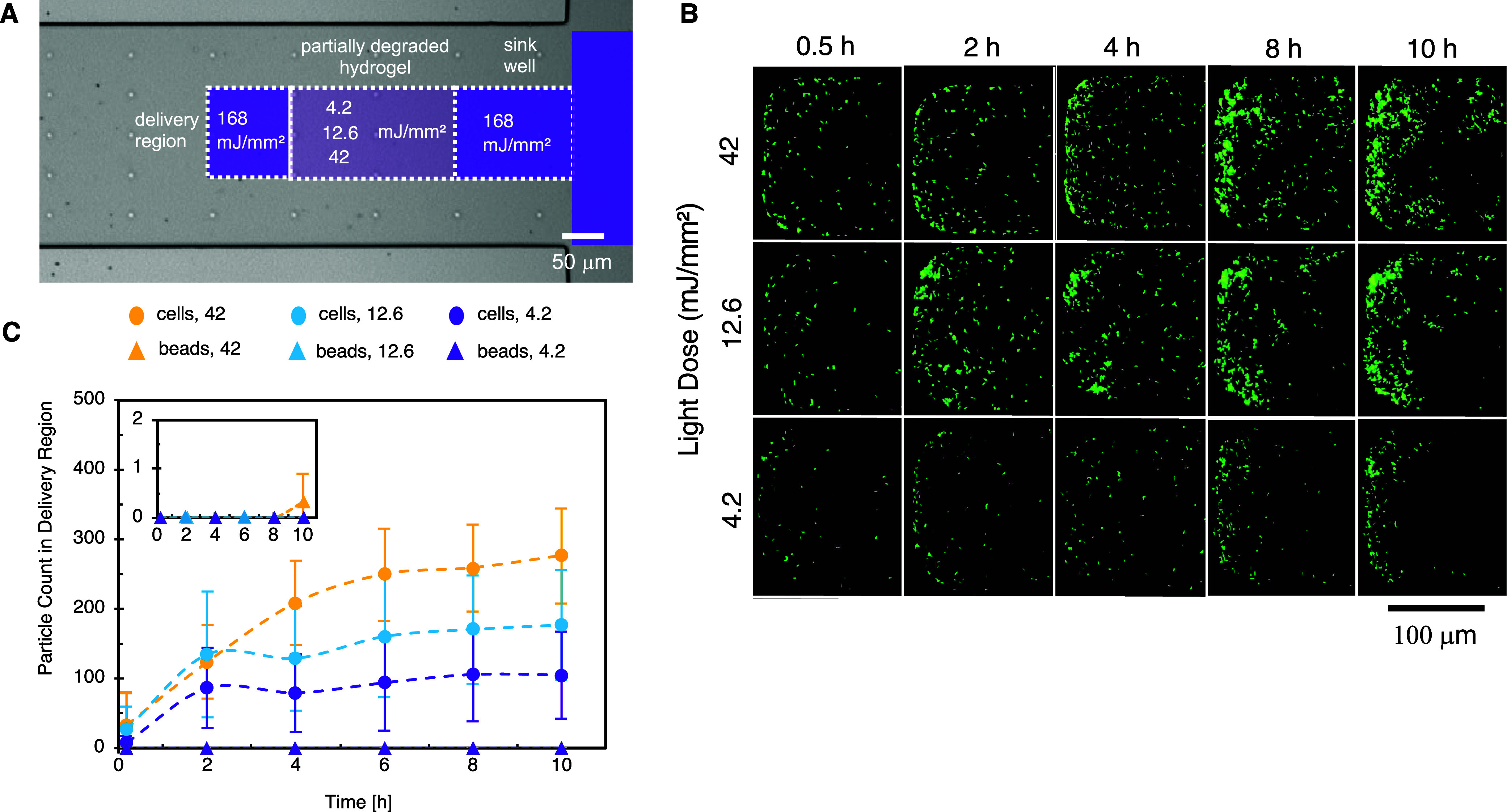
(A) Degradation map for selective, controlled release of *B. subtilis* into a fully degraded liquid “delivery” region. (B) Image montage of delivery region with time for each dose. (C) Cell and bead release profiles into the delivery region for 4.2, 12.6, and 42 mJ/mm^2^ dose in the partially degraded region. Inset: Zoomed-in region showing bead accumulation within the liquid delivery region (*n* = 3).

## 3 Conclusions

As PEG is cytocompatible but antiadhesive to bacteria, PEG-based hydrogels that are designed to provide tunable degradation—whether through light or another stimulus (temperature, pH, enzymatic, mechanical, etc)–are advantageous for controlled transport of live bacteria. In particular, the ability to guide cell motion, control mean cell speed, and modulate bacteria release profiles by tuning PEG degradation levels, as first demonstrated here, gives these hydrogels potential use as vehicles for controlled, localized *in vivo* delivery of therapeutic bacteria. While UV light is incompatible with bacteria and tissues, photonic nanoparticles can be added to these PEG-based hydrogels to cleave *o*-nitrobenzyl groups using cytocompatible, near-infrared light,^29^ which can provide local release of encapsulated cells within tissues and to a disease site, on-demand. At a more fundamental level, spatial control of degradation at the microscale combined with a microfluidic device that enables the generation of well-controlled chemical gradients offered insight into the movement of bacteria cells through these materials at varied stages of degradation. Here, we found distinct transitions in cellular motility patterns according to the degradation state of the hydrogel and found that bacteria learn to exploit paths of least resistance within partially degraded hydrogels to advance toward more favorable environments. Beyond bacteria therapeutics, such an experimental system can be applied to probe cell-hydrogel interactions in other applications that require bacteria transport (e.g., porous media analogs, synthetic microbial ecosystems), that require inhibition of bacteria infiltration (e.g., tissue engineering scaffolds, antifouling coatings), or both (microbial separation and isolation).

## 4 Materials and Methods

### 4.1 Design and Fabrication of Microfluidic Devices

All microfluidic master devices were designed and fabricated at the Center for Nanophase Materials Sciences at Oak Ridge National Laboratory. The microfluidic devices used in all studies were designed in Layout Editor. Devices consisted of two 4 mm circles (source and sink wells) connected by a 250 μm × 3000 μm rectangle channel (Figure S4A). 10 μm circular support pillars were placed 100 (*x*-direction) × 45.5 (*y*-direction) inside the plug region to prevent sagging of the PDMS during soft lithography. Sets of microfluidic masters were created using two processes. Silicon masters were produced using a combination of photolithography and reactive ion etching (RIE) or by photopatterning the negative photoresist SU-8 2015 (Kayakli Advanced Materials) on silicon wafers. Identical mask sets were used for optical contact lithography during both processes. A 5″ soda-lime chrome mask, precoated with chromium and photoresist (Nanofilm), was exposed with the desired patterns using a Heidelberg DWL 66 mask writer. The mask was developed in MF CD-26 developer (Shipley Company) for 1 min, rinsed with DI water, and dried with nitrogen. The mask was then etched in chromium etchant until the patterns were visibly cleared. The photoresist was removed in a heated bath of *N*-methyl-2-pyrrolidone (Dupont Electronic Materials International). The mask was rinsed in DI water and dried with nitrogen.

Silicon wafers (4-in. diameter ⟨100⟩ P-type, boron-doped to 10–20 Ω resistivity, 500–550 μm thick, single-side polished) were used as the substrate for microfluidic masters in all processes and were patterned on the polished side of the wafer. For silicon master fabrication with RIE, wafers were spin-coated with MicroPrime P20 adhesion promoter (Shin-Etsu MicroSi) to promote photoresist adhesion. NFR 016D2–55 cP (JSR Micro, Inc.), a negative photoresist, was spin-coated onto the wafers, soft-baked at 90 °C for 90 s, exposed to 365 nm light (50 mJ/cm^2^), and then postexposure baked at 115 °C for 90 s. Samples were developed using Microposit MF CD-26 developer (Shipley Company) for approximately 1 min or until visibly cleared, then rinsed with deionized water and dried with nitrogen. The wafers were etched to a depth of 8.3 μm (∼10 cycles) using a modified Bosch process (3 s polymer deposition, 10 s etch). SU-8 masters were created by first baking substrates on a hot plate at 180 °C for 10–30 min to dehydrate the wafers. Wafers were allowed to cool briefly and spin-coated at 3000 rpm with SU-8. Coated samples were soft-baked on a hot plate at 95 °C for 4 min, exposed in a contact aligner (150 mJ/cm^2^), and postexposure baked on a hot plate at 95 °C for an additional 5 min. Wafers were puddle-developed in SU-8 Developer (Kayakli Advanced Materials) for 3 min, rinsed with isopropanol, and dried with nitrogen. Etch depths or SU-8 feature heights were confirmed by measurement of select regions of the sample with a stylus profilometer (KLA Tencor).

### 4.2 Microfluidic Device Preparation

All microfluidic devices were made of PDMS. Silicon masters were first treated with trichloro (1*H*, 1*H*, 2*H*, 2*H*, -perfluorooctyl) silane (PFOS, Sigma-Aldrich) through a vapor deposition process to prevent PDMS adhesion to the microfluidic master during curing. The silicon wafers were placed in a glass 150 mm × 50 mm Petri dish with an Eppendorf tube cap filled with 20 μL of PFOS on a hot plate set at 100 °C. After 2 h, the hot plate was turned off, and the master device was left in the Petri dish overnight. 100 g total of PDMS was made with a 10:1 DOW SYLGARD 184 (Ellsworth Adhesives) Silicon elastomer base to catalyst ratio. The mixture was then degassed, poured over the silicon master device, degassed again, and cured at 80 °C for 2 h. Devices were then cut apart, and 4 mm biopsy punches (Electron Microscopy Sciences) were used to create wells on either side of the plug region. The device and a glass slide (25 mm × 75 mm × 1 mm) were sprayed with 70% isopropanol, dried with nitrogen, and then repeated with deionized water. Both the device and glass slide were then cleaned with oxygen plasma for 30 s using a PDC-001-HGP Plasma Cleaner (Harrick Plasma). The device and glass slide were then contacted together and heat-treated for 10 min at 80 °C. A vacuum pump (Pittsburgh Automotive, 3 CFM) and desiccator (SP Bel-Art, 0.20 cu.ft.) were used to purge the air out of the device for 20 min. At this point, devices were ready for perfusion of the *pd*-PEG precursor solution within the channel. A picture of a sample microfluidic device setup is provided (Figure S8).

Prior to preparing the *pd*-PEG precursor solution, phosphate-buffered saline (PBS) buffer was made in-house by combining 1,567 mg NaH_2_PO_4_, and 187 mg and Na_2_EDTA (Sigma-Aldrich) to 90 mL in ultrapure water and adjusting the pH to 8.0. The PBS solution was sterile-filtered and stored at room temperature prior to use. The PEG-*o*-NB-DA macromer was synthesized in-house; a detailed description of its synthesis and an H^1^ NMR spectra of the exact PEG-*o*-NB-DA product used here is available in Fattahi et al. (Scheme S1 and Figure S1).^24^ To prepare the precursor solution, 25 μL of PBS was combined with 11.2 μL of PEG-*o*-NB-DA in an Eppendorf tube and mixed thoroughly. 13.8 μL of Pentaerythritol tetra­(mercaptoethyl) polyoxyethylene (PEGTT, MW 5.0 kDa, NOF America Corporation) was then added and mixed. Then 10 μL of the solution was quickly pipetted into the 4 mm wells, allowing for perfusion of the precursor throughout the channel. The device was incubated at room temperature for 30 min to allow for hydrogel formation, then 20 μL of TSB (30 g/L, Sigma-Aldrich) was added to one well, and 20 μL of PBS on the other side. This was left covered 24 h before proceeding. After this, the 4 mm biopsy punch was used to clear the hydrogel out of each well and then the wells were washed with 20 μL of PBS.

### 4.3 Chemical Gradient Characterization and Diffusivity Calculation

Fluorescent chemical gradients were produced using a 6.1 mM solution of Alexa Fluor 594 NHS ester (Thermo-Fisher Scientific) in PBS instead of TSB solution. 20 μL of this solution was added to the source well, and 20 μL of PBS only was added to the sink well. Fluorescent images were taken down the channel every hour for 48 h using a Cytation 5 (BioTek) and a 20× objective. Images were montaged together to create a single composite image displaying the entire channel. Intensity profiles down the plug region were then measured using ImageJ software, normalized, and plotted for various time points. To determine the diffusion coefficients (*D*_eff_) of this molecule in the hydrogel, transient fluorescent intensity profiles between 0 and 48 h were analyzed at three different positions within the channel ((*x*/*L)* = 0.4, 0.5, and 0.6) using Fick’s second law simplified with an early time approximation (*I*_*x*_(*t*)/*I*_max_ ≤ 0.6)^43^

$$\frac{I_{x}\left(t\right)}{I_{\max}}={4\left(\frac{D_{\mathrm{eff}}t}{\pi x^{2}}\right)}^{0.5}$$

where *I*_*x*_(*t*) is the fluorescent intensities in the channel at a given position at time *t*, *I*_max_ is the fluorescent intensity at that position at the final time point (*t* = 48 h), and *x* corresponds to the position in the channel. As diffusion is independent of channel position in these regions, the *D*_eff_ values measured at the different channel positions were averaged together.

### 4.4 Photodegradation of Hydrogel in Device Channels Using Patterned Illumination

After deposition in channels, *pd*-PEG hydrogels were exposed to user-defined 365 nm light patterns using a Polygon 400 patterned illumination tool (Mightex Systems) attached to an Olympus BX51 upright microscope (Figure S4A). This tool allows for control of light intensities ranging from 0.7–7.0 mW/mm^2^ and millisecond control of exposure times at ∼9 μm pattern resolution. Prior to patterning, the Polygon tool was calibrated using a calibration mirror as described in Fattahi et al.^39^ All hydrogel degradations were performed using 60% light intensity (4.2 mW/mm^2^) at a 20×, NA 0.5 objective. For patterning of the degradation ladder, micropatterned rectangles 110 μm (*x*-direction) by 101 μm (*y*-direction) were used. This pattern corresponds to the distance between one column of support pillars in the *x*-direction and three rows of support pillars in the *y*-direction (dashed white line, Figure S4B). To ensure that degradation was achieved between different exposure regions, adjacent exposure rectangles overlapped 10 μm in the *x*-direction, corresponding to the diameter of a support pillar. Exposure times of 0.5, 1, 3, 10, and 40 s were chosen, generating respective doses of 2.1, 4.2, 12.6, 42, and 168 mJ/mm^2^. The latter was chosen as the upper limit for dose, as this was the dose used for complete hydrogel degradation and reverse gelation in prior studies by Fattahi et al.^24^ The 2.1 mJ/mm^2^ region had one exposure rectangle, and both 4.2 and 12.6 mJ/mm^2^ exposure regions had two exposure rectangles. The 42 mJ/mm^2^ region was exposed from the plug region up to the second set of supporting pillars. To ensure bacteria had access to this region, rectangles (165 μm × 260 μm) were exposed where the plug region met the edge of the well using a 168 mJ/mm^2^ dose, which also degraded any residual hydrogel remaining after removing hydrogel from the wells using the 4 mm biopsy punch. After exposing the hydrogel to this degradation pattern, the sink well was briefly rinsed with 20 μL PBS to remove degraded PEG byproducts. For experiments involving the selective delivery of bacteria, a section of hydrogel ∼130 μm into the channel was fully degraded using 168 mJ/mm^2^ doses, up to the second column of supporting pillars. A second 220 μm × 101 μm region of partially degraded hydrogel was exposed to doses of 4.2, 12.6, or 42 mJ/mm^2^. Finally, a 110 μm × 101 μm liquid delivery region was exposed to a 168 mJ/mm^2^ dose for reverse gelation (Figure S4C).

### 4.5 Hydrogel Staining

To visualize partially degraded hydrogels within the device, a 40 μM solution of fluorescein-5-maleimide was prepared by adding 4 μL of 10 mM fluorescein-5-maleimide (Thermo-Fisher Scientific) in DMSO to 1 mL of PBS (pH 8.0). 20 μL of this solution was then added to each well for 2 h. Each well was then washed with 20 μL of PBS to remove unbound fluorophore, and the hydrogel was imaged using a Nikon TI-E inverted fluorescent microscope with a FITC filter.

### 4.6 Bacteria Chemotaxis through Partially Degraded Hydrogels

*B. subtilis* strain 1A1135 (Bacillus Genetic Stock Center, Columbus, OH, United States) modified to express green fluorescent protein (GFP) and for spectinomycin resistance, was used in all chemotaxis experiments. *B. subtilis*-GFP was stored in 50% glycerol stocks at −80 °C until use. For chemotaxis experiments, *B. subtilis*-GFP was first cultured in TSB with 100 μg/mL spectinomycin (Sigma-Aldrich) at 32 °C (215 rpm) for 24 h. Optical densities at 600 nm (OD_600_) were then measured from a 100 μL sample of a *B. subtilis*-GFP culture solution using an Epoch2 microplate reader (BioTek) in 96-well format. Cultures were diluted with PBS to an OD_600_ of 0.01 for further use. To introduce *B. subtilis*-GFP into the device, 19 μL of PBS and 1 μL of bacteria solution (OD_600_ = 0.01) were added to the sink well, and a 20 μL solution of TSB (16 μL PBS and 4 μL 30 g/L TSB) was added to the nutrient source well. The region of interest containing degraded hydrogel was monitored with time-lapse fluorescence microscopy (TLFM) using a Nikon Ti-E inverted fluorescent microscope with a 20×, NA 0.45 objective, a FITC filter set (gain 25.6× and exposure 100 ms), and an X-Cite illumination source immediately after *B. subtilis*-GFP was introduced into the sink well. After this, a glass slide was placed over the top of the device to cover the source and sink wells and prevent evaporation of the liquid and drying of the hydrogel. For experiments involving *B. subtilis*-GFP in red fluorescent polystyrene beads, 18 μL of diluted TSB was added to the source well, and 18 μL of PBS was added to the sink well. 1 μL of *B. subtilis*-GFP at an OD_600_ 0.01 and 1 μL of a 0.025 weight/volume % solution of red fluorescent polystyrene amine-modified latex beads (Sigma-Aldrich, 2.5% solids) in PBS were added to the sink well for a final *B. subtilis* OD_600_ of 0.005 and a 0.00125% solid solution, which was approximately equivalent to a 10:1 bead to cell ratio (Figure S7). After the introduction of the mixed solution, a glass slide was placed over the top of the device, and the rectangular delivery region was monitored using TLFM. Images of the bacteria and fluorescent beads were taken using the same microscope, objective, and illumination source as stated before for the degradation ladder. A FITC filter set (gain 25.6× and exposure 100 ms) was used for the bacteria and a TRITC filter set (gain 9.3×, exposure 40 ms) was used for the fluorescent beads.

### 4.7 Image Analysis

Images and movies were viewed and edited in FIJI (ImageJ2). Individual cell counts were measured using the FIJI multipoint tool. All mean speeds and mean directional changes, the latter defined as the angle value (in radians) between succeeding links of a track, averaged over the entire track, were calculated using the TrackMate FIJI plug-in. This plug-in provides *x*- and *y*-coordinates for each bacteria detected in each frame. The initial coordinates were set to zero to calculate the individual tracks of bacteria and to provide a comparison of tracks between individual cells. For cell migration maps, the final *x*- and *y*-locations for each track after the 24 h chemotaxis experiments from Trackmate were binned within a 7 × 7 grid. Bin sizes within the grid were created according to the minimum and maximum *x* and *y*-locations traveled by cells in each population. The binned bacteria locations created a histogram representing the migration of the entire population of bacteria. Bicubic interpolation of this data was then used to create the cell migration maps. A white cross at the center of the image indicates the initial position of all bacteria.

### 4.8 Statistical Analysis of Data

All statistical analysis was done using SAS Institute Inc. Statistical significance was determined using student *t* tests and Tukey’s test; an α value less than 0.05 was considered statistically significant. All calculated means and standard deviations are based on a minimum of *n* = 3 independent replicates.

## Supplementary Material















## Data Availability

The data used in this study and the Python code used to create the cell migration maps are available upon reasonable request.

## Notes

Simulated concentration profile of Alexa Fluor 594 dye (Figure S1); Difco tryptone (Figure S2); and glucose (Figure S3); layout editor design of the microfluidic device and degradation patterns used (Figure S4); normalized intensity profiles for nondegraded hydrogel in the *x*- and *y*-direction (Figure S5); line degradation of hydrogel with individual bacteria tracks (Figure S6); red-green fluorescence image of fluorescent bead to bacteria ratio (10:1 ratio) loaded into sink well for selective delivery experiment (Figure S7), and top view of microfluidic device set up on the microscope (Figure S8) (PDF)
*B. subtilis* cells are unable to penetrate into fully intact hydrogel interface (Supp. Mov. 1) (AVI)
*B. subtilis* movement through the degradation ladder (Supp. Move. 2) (AVI)
*B. subtilis* movement through three lines of different degradation (Supp. Mov. 3) (AVI)
Time lapse of selective *B. subtilis* delivery using 42 mJ/mm^2^ dose in partial degradation region (Supp. Mov. 4) (AVI)
Time lapse of selective *B. subtilis* delivery using 12.6 mJ/mm^2^ dose in partial degradation region (Supp. Mov. 5) (AVI)
Time lapse of selective *B. subtilis* delivery using 4.2 mJ/mm^2^ dose in partial degradation region (Supp. Mov. 6) (AVI)
