# Supplementary material for: Photodegradable Hydrogel Matrices for Spatiotemporal Control of Bacteria Transport and Delivery
Source: ACS Appl Mater Interfaces. 2025 Sep 2;17(37):51919–30. doi: 10.1021/acsami.5c14670 (PMC12447405; doi:10.1021/acsami.5c14670)
Supplement: Supplementary file 1 [file am5c14670_si_001.pdf]

SUPPORTING INFORMATION

Photodegradable Hydrogel Matrices for Spatiotemporal Control of Bacteria Transport and Delivery

Jeffrey A. Reed[1], Scott T. Retterer[2], Ryan R. Hansen[1, †].

[1]Tim Taylor Department of Chemical Engineering, Kansas State University, Manhattan, KS 66506, USA

[2]Center for Nanophase Material Sciences/Biosciences Division, Oak Ridge National Laboratory, Oak Ridge, TN 37830, USA

[†]Corresponding author: Ryan R. Hansen, Address: 1017 Durland Hall, Manhattan, KS, 66506, United States of America. Email: rrhansen@ksu.edu.

Page		Description
**S3**	**S1.0**	Estimation of the primary carbon/nitrogen source in tryptic soy broth (TSB) through the hydrogel
**S4**	**Figure S1**	Passive diffusion transport model of Alexa Fluor 594 through the hydrogel in the microfluidic channel
**S4**	**Figure S2**	Passive diffusion transport model of Difco tryptone through the hydrogel in the microfluidic channel
**S5**	**Figure S3**	Passive diffusion transport model of glucose through the hydrogel in the microfluidic channel
**S5**	**Figure S4**	Layout editor design of the microfluidic device and degradation patterns used
**S6**	**Figure S5**	Normalized intensity profiles for non-degraded hydrogel in the x- and y-direction
**S7**	**Figure S6**	Line degradation of hydrogel with individual bacteria tracks
**S8**	**Figure S7**	Red-green fluorescence image of fluorescent bead to bacteria ratio (~10:1 ratio) loaded into sink well for selective delivery
**S9**	**Figure S8**	Top view of microfluidic device set up on the microscope
**S10**	**Supporting Information References**	

S1.0 Estimated tryptic soy broth (TSB) diffusion coefficient

TSB is a complex mixture of casein/soy peptones, sodium chloride, and natural sugars, with the main carbon and nitrogen sources required for growth coming from glucose and tryptone (digest of casein), respectively.[1] The diffusion coefficient of glucose has previously been measured through similar PEG hydrogels (1.29 x 10^{-6} cm^2/s).[2] Difco tryptone is a digest of casein protein and is the primary nitrogen source in TSB; its peptide molecular weight breakdown is given on page 61 of the Thermo-Fisher technical guide to peptones, supplements, and feeds.[1]

To estimate the diffusion coefficient (D_0) for each peptide molecular weight range in water, the Stokes-Einstein equation was used:[3]

$$D_o = \frac{k_b T}{6\pi\eta R_h} \qquad (1)$$

where k_b is the Boltzmann constant, T is the absolute temperature, and η is the dynamic viscosity of the solvent (water), and R_h is the average hydrodynamic radius for each peptide molecular weight range. Equation 5 in Wilkins et al.[4] was used to calculate R_h for each unfolded/denatured peptide for each molecular weight range shown as:

$$R_h = (2.21 \pm 1.07) N^{0.57 \pm 0.02} \qquad (2)$$

Where N is the number of residues in the polypeptide chain. While this equation is in terms of amino acid residues instead of molecular weight, the average weight of an amino acid (110 Da) is used to estimate the number of residues. The middle value of each peptide range (125 Da for <250 Da and 1,250 for 500-2,000 Da, etc.) was used for each size. The weighted average diffusion coefficient ($\overline{D_0}$) was calculated with Equation 3:

$$\overline{D_0} = \sum\left(\frac{M_i}{100} * D_i\right) \qquad (3)$$

where M_i is the mass fraction for each size range. With this, $\overline{D_0}$ was calculated to be 5.09x10^{-6} cm^2/s. Assuming an average reduction in diffusivity of 88% through the hydrogel, which has been measured with other small molecules through these hydrogels,[5] the effective diffusion of Difco Tryptone (D_{eff}) through the hydrogel is estimated to be 6.11 x 10^{-7} cm^2/s. With these diffusion coefficients, a Python script was created to model passive diffusive transport of glucose and tryptone components through the microfluidic device channel. The model uses Fick's second law:

$$\frac{\partial C_a}{\partial t} = D_{eff}\left(\frac{\partial^2 C_a}{\partial x^2}\right) \qquad (4)$$

and assumes constant concentrations of each component at the source well (x=0 μm) and sink well (x=3000 μm) as boundary conditions. The resulting concentration profiles across the channel over 48 hrs are provided for Alexa 594 dye (Fig. 2A below) which matches experimental data (Fig. 2 in main manuscript), for tryptone (Fig. 2B below), and finally for glucose (Fig. 2C below). The model shows that 24 hrs was sufficient time for glucose and tryptone to form a linear, steady-state gradient across the hydrogel in the actual chemotaxis experiments.

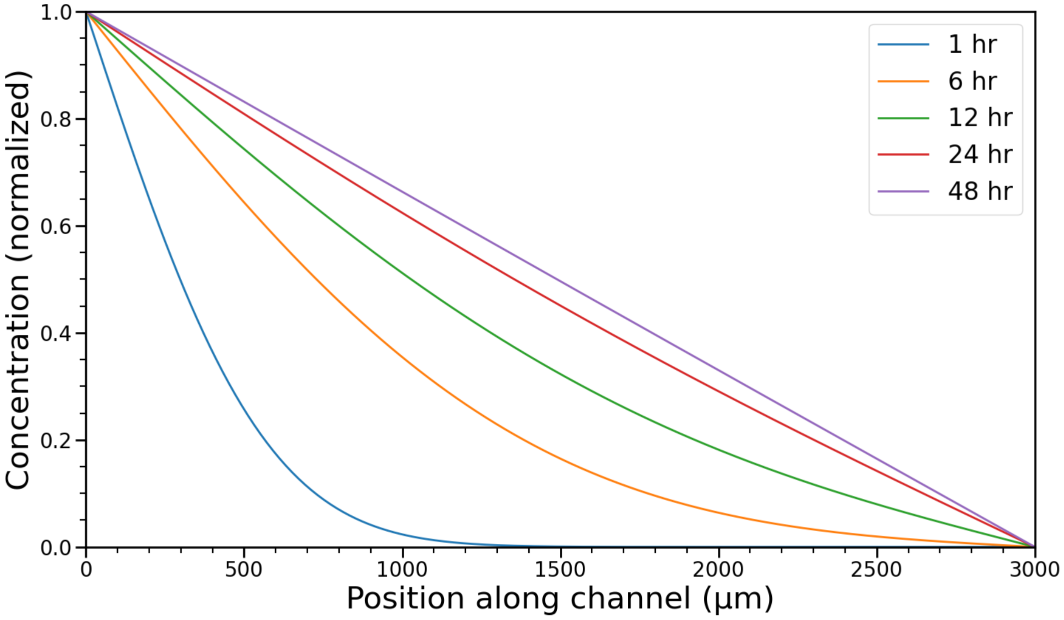

Figure S1. Simulated concentration profile of Alexa Fluor 594 dye through microfluidic channel according to Fick's Second Law.

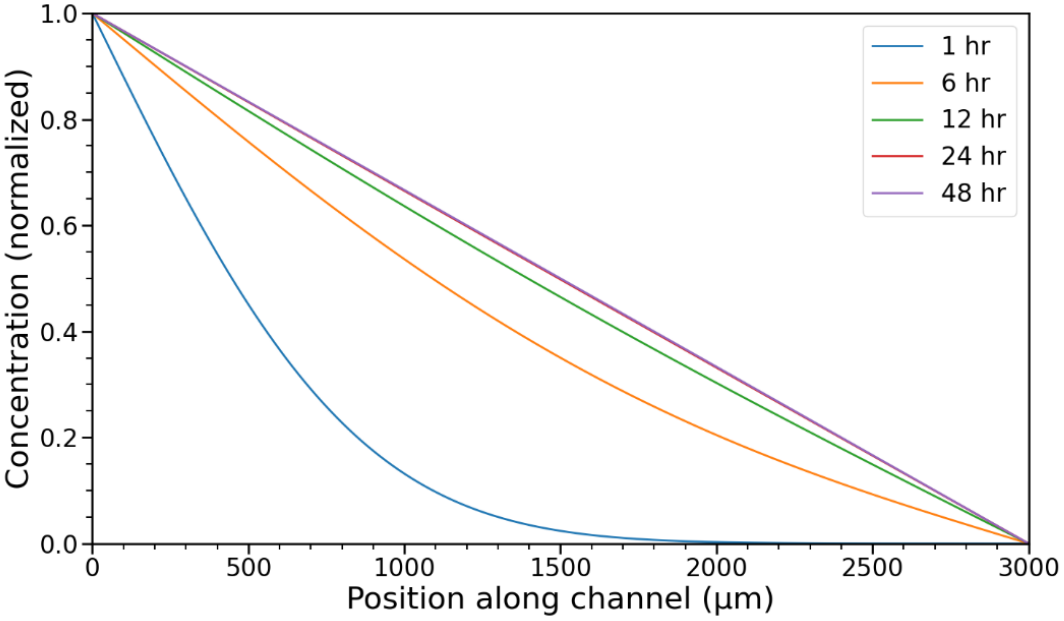

Figure S2. Simulated concentration profile of difco tryptone through microfluidic channel according to Fick's Second Law.

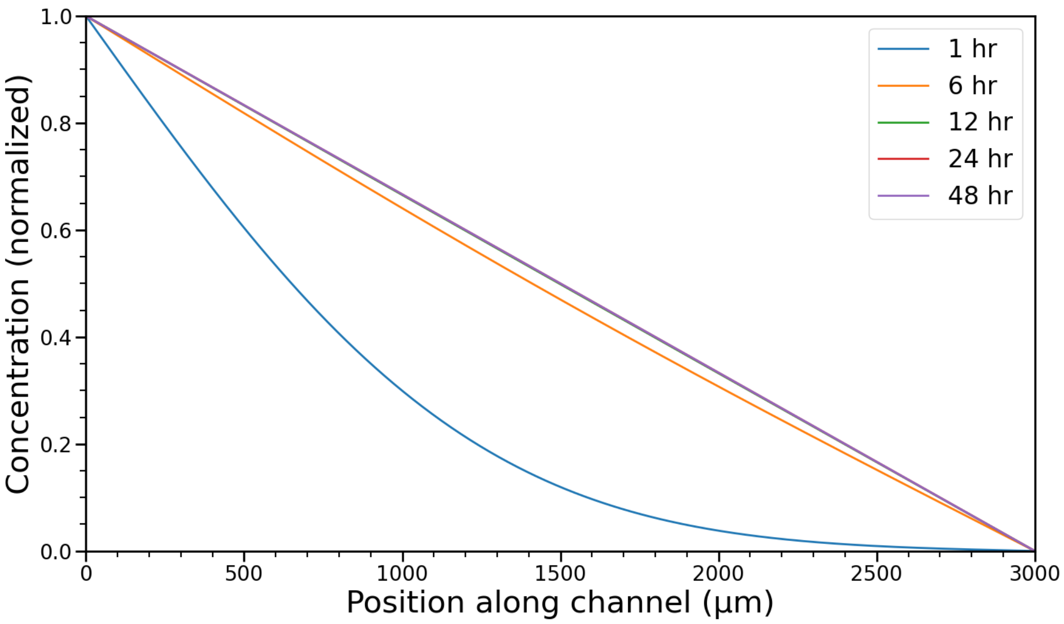

Figure S3. Simulated concentration profile of glucose through microfluidic channel according to Fick’s Second Law.

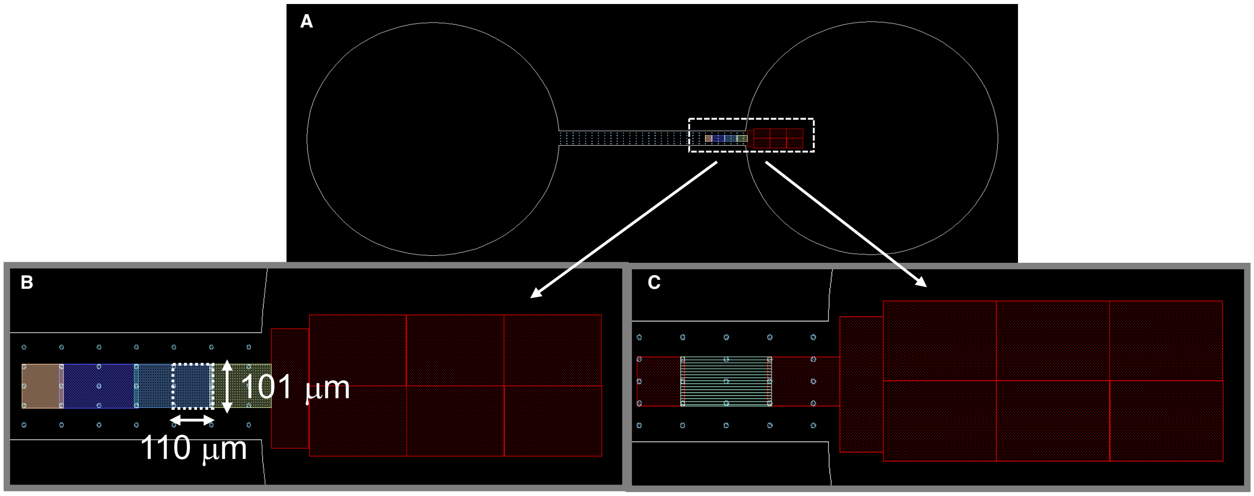

Figure S4. (A) CAD design of entire microfluidic device in layout editor. Boxes show areas exposed with 365 nm ultraviolet light. Zoomed in view of the region of interest for the (B) degradation ladder experiments and (C) selective delivery experiment.

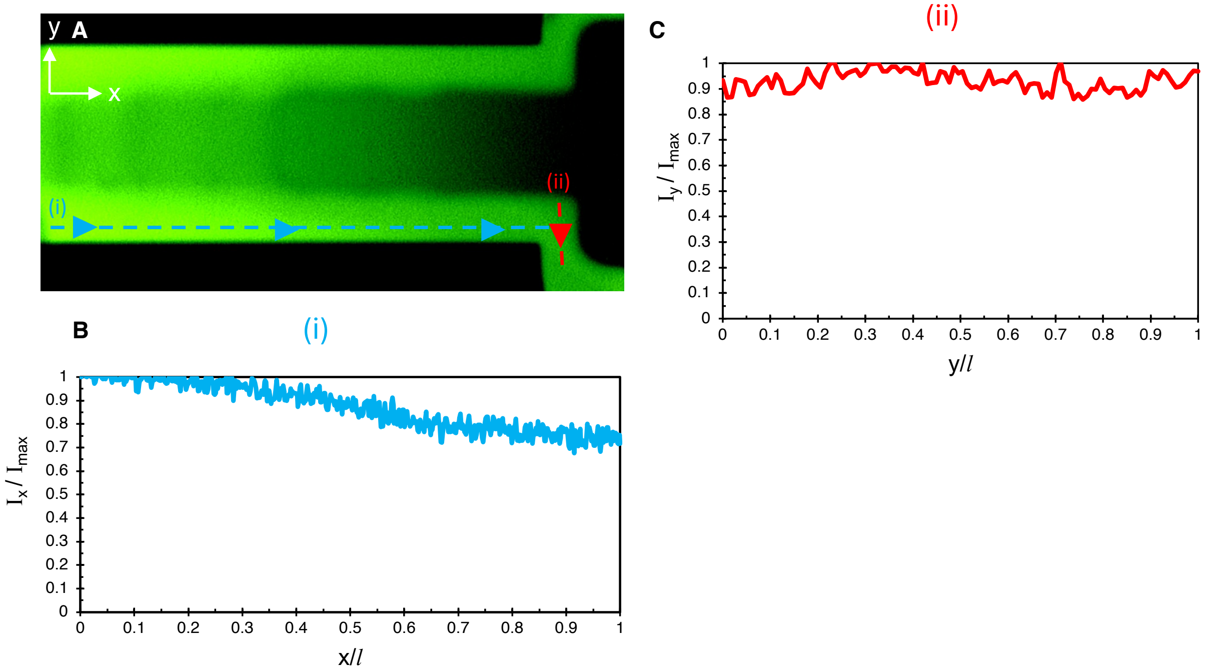

Figure S5. (A) Image of non-degraded hydrogel regions after staining with Fluorescein-5-Maleimide. Normalized intensity profile in the (B) x-direction and (C) y-direction.

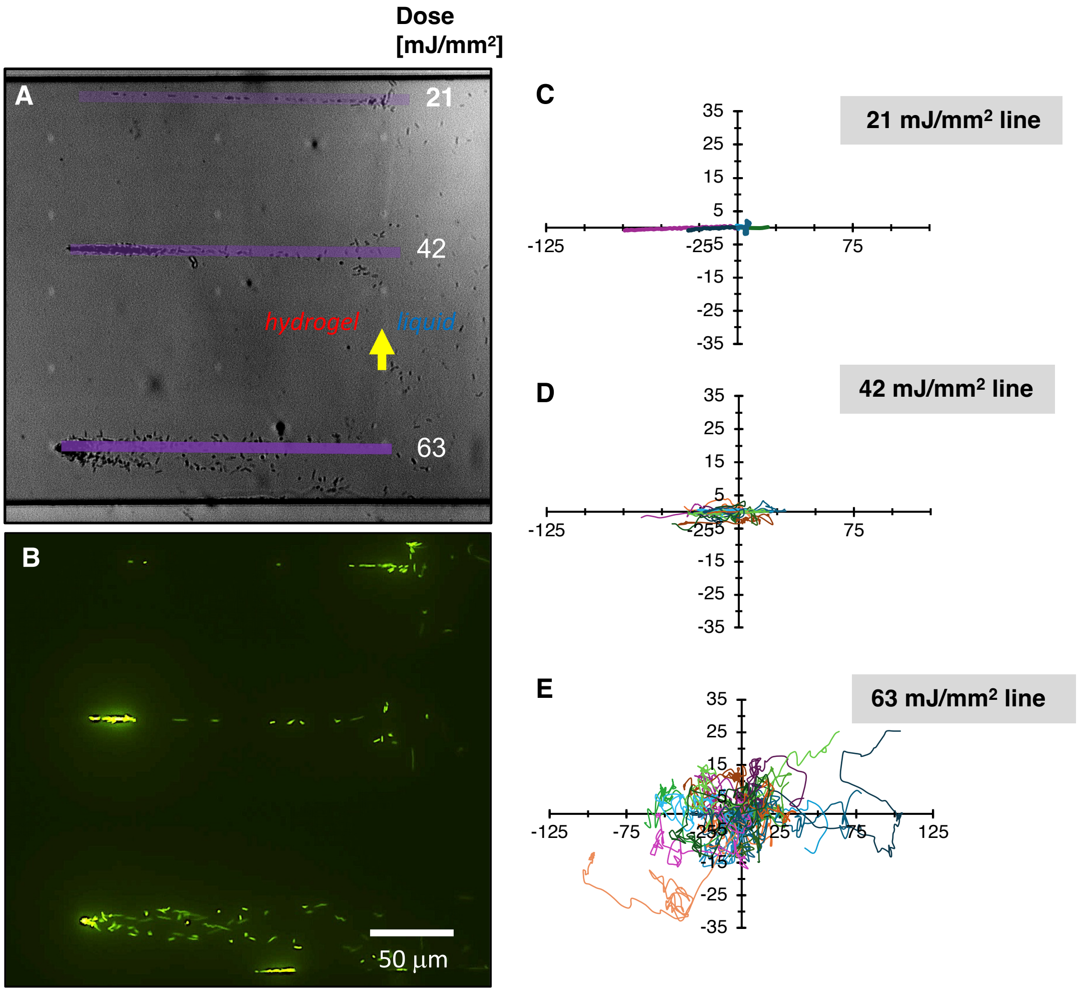

Figure S6. Image of (A) degradation map and (B) fluorescent image showing bacteria protrude into partially degraded regions. Individual cell trajectories and rose plot for (C) 21 mJ/mm^2 line, (D) 42 mJ/mm^2 line, and (E) 63 mJ/mm^2 line.

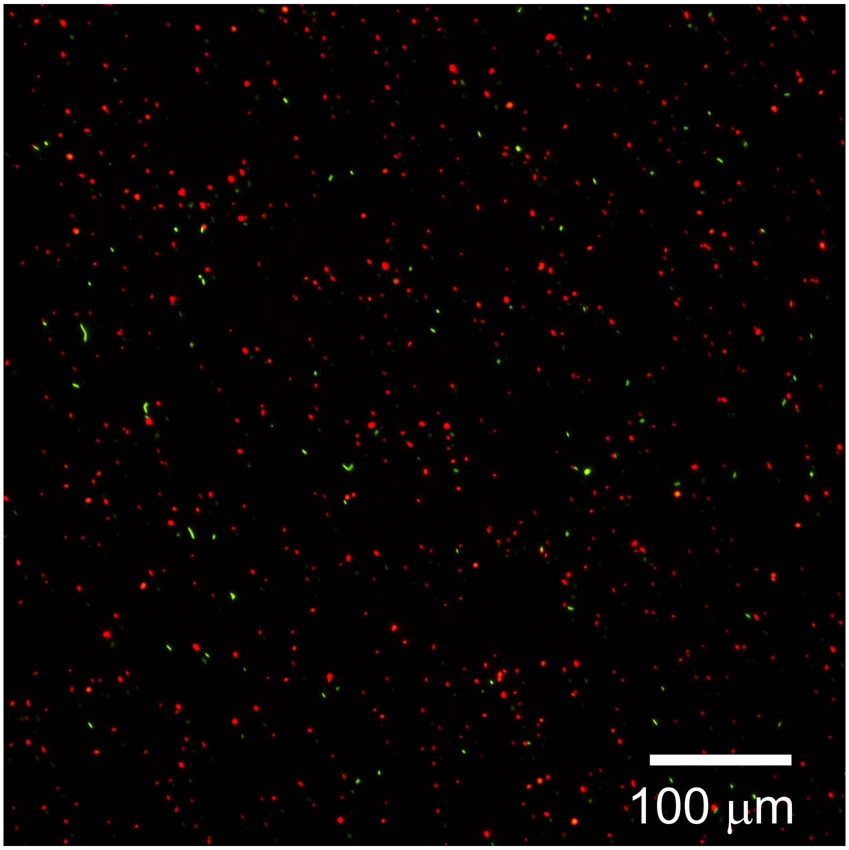

Figure S7. Red-green fluorescent image of B. subtilis-GFP cells (green) mixed with 1 mm diameter polystyrene beads (red) at a ~10:1 bead:cell ratio. This solution was added to the sink well.

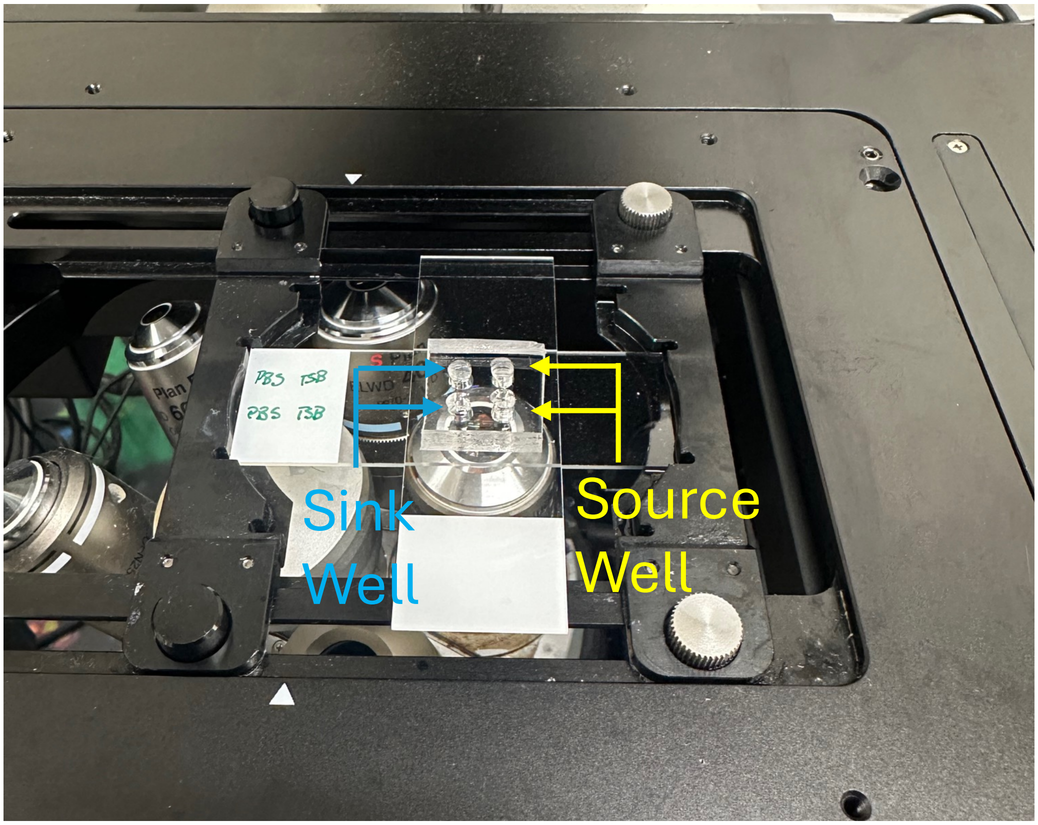

Figure S8. Microfluidic device set up. Each PDMS device contained two separate sink and source wells connected by a microfluidic channel.

Supporting Information References

(1) Thermo Fisher, S. Peptones, Supplements and Feeds: Technical Reference Guide. 1 ed.; Thermo Fisher Scientific: Waltham, MA, USA, 2019; p 61.

(2) Cavallo, A.; Madaghiele, M.; Masullo, U.; Lionetto, M. G.; Sannino, A. Photo-crosslinked poly (ethylene glycol) diacrylate (PEGDA) hydrogels from low molecular weight prepolymer: swelling and permeation studies. *Journal of Applied Polymer Science* **2017**, *134* (2). DOI: 10.1002/app.44380.

(3) Axpe, E.; Chan, D.; Offeddu, G. S.; Chang, Y.; Merida, D.; Hernandez, H. L.; Appel, E. A. A Multiscale Model for Solute Diffusion in Hydrogels. *Macromolecules* **2019**, *52* (18), 6889-6897. DOI: 10.1021/acs.macromol.9b00753.

(4) Wilkins, D. K.; Grimshaw, S. B.; Receveur, V.; Dobson, C. M.; Jones, J. A.; Smith, L. J. Hydrodynamic Radii of Native and Denatured Proteins Measured by Pulse Field Gradient NMR Techniques. *Biochemistry* **1999**, *38* (50), 16424-16431. DOI: 10.1021/bi991765q.

(5) Fattahi, N.; Reed, J.; Heronemus, E.; Fernando, P.; Hansen, R.; Parameswaran, P. Polyethylene glycol hydrogel coatings for protection of electroactive bacteria against chemical shocks. *Bioelectrochemistry* **2024**, *156*. DOI: 10.1016/j.bioelechem.2023.108595.
